# A practical development protocol for evidence-based digital integrative arts therapy content in public mental health services: digital transformation of mandala art therapy

**DOI:** 10.3389/fpubh.2023.1175093

**Published:** 2023-09-28

**Authors:** Hyungsook Kim, Yoonyoung Choi

**Affiliations:** ^1^HY Digital Healthcare Center, Hanyang University, Seoul, Republic of Korea; ^2^Department of Cognitive Sciences, School of Intelligence, Hanyang University, Seoul, Republic of Korea; ^3^Graduate School of Public Policy, Hanyang University, Seoul, Republic of Korea; ^4^Department of Integrative Arts Therapy, Graduate School, Dongduk Women's University, Seoul, Republic of Korea

**Keywords:** digital public health, digital healthcare, evidence-based digital content, integrative arts therapy, digital transformation, digital integrative arts therapy, structured mandala coloring

## Abstract

**Introduction:**

The fundamentals of digital transformation include the conversion of the traditional method into a digital format to develop a standardized system that collects, analyzes, and processes quantitative data. This study aims to provide a comprehensive understanding of the development process and key elements of evidence-based digital integrative arts therapy.

**Methods:**

The “Digital Mandala” service in the “Mental Health App” produced as part of a national public mental health project for personalized depression management is adopted to explain how to convert the existing mandala art therapy into digital format. A living lab approach has been applied, which can be used to address the nation's mental health challenges by promoting collaboration, innovation, and evidence-based solutions.

**Results:**

Evidence-based digital content requires evidence that covers the structural process, the effects of existing methods, and the components and meanings of each detailed scene. In this section, we provide five stages of the development process, including preliminary research, design, development, commercialization, and advancement. Consequently, clinical elements, integrative arts therapy features, and data factors are defined as the key principles of evidence-based digital integrative arts therapy.

**Discussion:**

Based on the data factors found in this study, it will be possible to create an evaluation dataset of digital integrative arts therapy content for managing depression. Additionally, the large-scale public data can be analyzed through artificial intelligence technology, which is expected to be used as a basis for deriving significant results in a new form, going further than the existing evaluation method. This research is significant because it establishes the foundation for digital transformation in the field of art therapy for public mental health services and investigates its potential.

## 1. Introduction

Innovation in the medical field is accelerating since the frontier technology that forms the foundation for the Fourth Industrial Revolution [i.e., big data, artificial intelligence, Internet of Things (IoT), Cloud] has begun to be applied to the healthcare arena (1). The new paradigm of modern healthcare services has brought forward the concepts of digital healthcare and wellness, which combine advanced medical technology and information and communication technology (ICT), while changing the disease-centered treatment into a health-centered prevention and management system (2). According to the U.S. Food and Drug Administration (3), digital health encompasses diverse concepts, including mobile health (mHealth), health information technology (IT), wearable devices, telemedicine, telehealth, and personalized medicine. Digital mental health applications are a specialized component of the rapidly expanding app market with the potential for development and testing (4). Smartphones, social networks, and internet application technology have changed how we communicate and provide innovative methods to monitor health and wellbeing, allowing us greater access to information (3).

The Digital Wellness Service (DWS), which introduced after the development of ICT, encompasses digital technologies or content for our body and mind to promote a healthy and balanced life (5). It provides a predictable and customized prevention and health management system based on big data and analyzes and reviews the generated health-related data. According to recent studies, digital interventions have increased opportunities in the mental healthcare domain (6). Arts therapy related to digital interventions is also being conducted (7–10). However, there is little evidence-based guidance despite the availability of numerous digital psychotherapeutic approaches (11). In contrast to research-based digital interventions, well-known mHealth apps that are accessible in major app stores frequently do not provide evidence on the theoretical or empirical basis of their content (12–15).

The development of DWS requires a systematic approach and not just the use of digital media. A new strategy is required to innovate based on digital technology that utilizes ICT, such as big data, artificial intelligence (AI), and cloud computing. The core of digital transformation is the conversion of the traditional operation method into a digital format to digitalize and establish a standardized system that collects, analyzes, and processes quantitative data. Digital transformation refers to quantifying unquantified areas. It can facilitate quantitative evaluation in arts therapy, which was previously qualitative in nature. Digital conversion through data factor derivation can provide quantitative analyses in qualitative fields. It meets the needs of digital individuals and can be very useful for mental health.

This study aims to identify evidence-based digital transformation methods by presenting a practical development protocol for the “Digital Mandala” service in the “Mental Health App” produced as part of a national public mental health project for personalized depression management. Living lab approaches have been applied to the development of digital integrative arts therapy. This article provides an in-depth description that details how traditional mandala art therapy was transformed into a digital form (RQ1). Additionally, it clarifies the core components of evidence-based digital integrative arts therapy by presenting the evidence supporting the elements of each scene and why the content was organized this way during the digital transformation process (RQ2). Ultimately, this study emphasizes the importance of seizing the opportunities that the digital world offers in the future and focuses on mental health.

## 2. Methods and tools

### 2.1. Digital Mandala in public Mental Health App services

The Seoul Metropolitan Government has introduced a pilot program for the “Mental Health App” service, which is being tested by 500 young individuals participating in the “Youth Mental Health Management” support project (16, 17). The development of the Mental Health App was a collaborative effort between HY Digital Healthcare Center (HY-DHC), which includes professionals such as psychiatrists and arts therapy specialists, and Vantage Digital Point (VDP Labs Inc.). The authors and participating institutions are affiliated with the same research unit. This project represents a cluster of collaborations between industry, research, government, and healthcare institutions.

The Mental Health App provides user-customized digital content to diagnose mental health and prompt recovery from depression (18, 19). As a digital public service, this software includes digital psychological tests for depression (Center for Epidemiological Studies Depression Scale: CES-D-10 and PHQ-9), anxiety (Generalized anxiety Disorder Scale: GAD-7), and sleep disorder (Insomnia Severity Index: ISI-K4). Users can review the test results based on the self-screening, and an algorithm will recommend three types of personalized digital content to manage depression. The test results for depression can be classified as mild (low depression), moderate (moderate depression), or major (high depression). The content comprises the “Physical Activity Game” to improve muscle strength and cardiorespiratory capacity, the “Finding Blue Game” as a cognitive and emotional screening service and the “Digital Mandala” as a digital integrative arts therapy. These contents are activated according to the user's depression test results.

**Figure 1:**
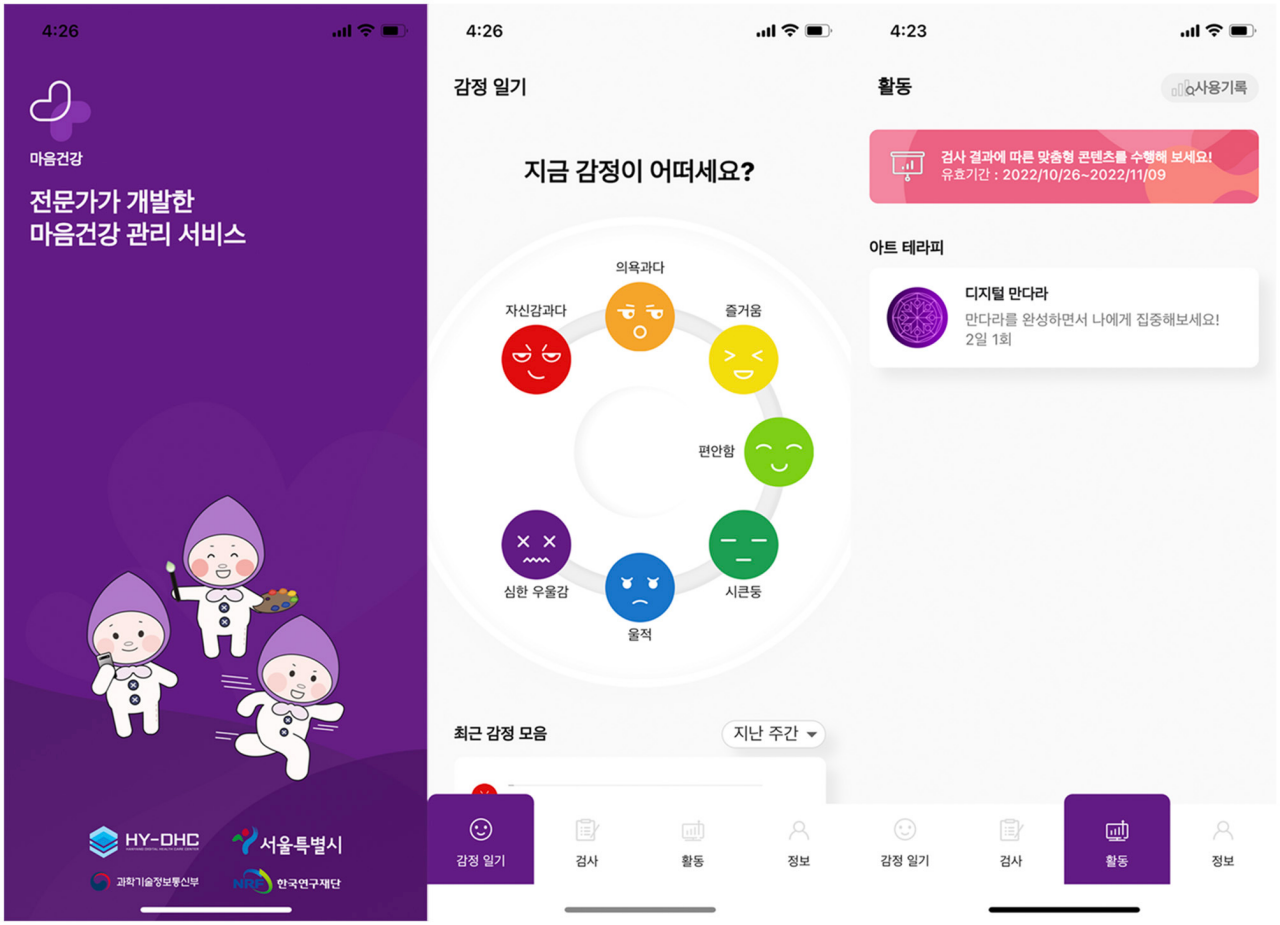
Mental Health App.

This study focuses specifically on providing a detailed description of a practical development protocol for evidence-based digital content. In this regard, as a research tool, we selected the Digital Mandala service to examine the digital transformation method and explore the evidence-based elements of digital integrative arts therapy. Figure 1 depicts the Mental Health App's splash screen, emotion diary, and recommendation screen of the Digital Mandala.

### 2.2. Study design and digital transformation process

This study provides comprehensive and thorough information and explanations about the digital transformation method and content development process of mandala art therapy (RQ1) and the core elements of evidence-based digital integrative arts therapy (RQ2). In this project, we have implemented the Digital Mandala service, which converted the traditional experiential mandala art therapy technique, known as structured mandala coloring, into a digital format. The digital transformation process and content development protocol involved the following procedures: First, an analysis of the session operating structure of traditional mandala art therapy was conducted. Second, the core elements necessary for the digital transformation of mandala art therapy were analyzed and presented. Third, evidence-based digital integrative arts therapy content was designed and implemented in digital form. Table 1 provides the study design of evidence-based digital content development framework.

**Table 1:** Study design for the evidence-based digital integrative arts therapy content.

**Digital transformation of mandala art therapy**
**Evidence-based digital integrative arts therapy content development framework**
**RQ1. The digital transformation method and content development process of mandala art therapy**
- The structuring existing mandala art therapy - 5 stages of digital content development
**RQ2. The core elements of evidence-based digital integrative arts therapy content**
- Clinical factors - Integrative arts therapy factors - Data factors

## 3. Results

### 3.1. A practical development protocol for digital transformation of mandala art therapy

#### 3.1.1. The structuring existing mandala art therapy

Mandala is a magic circle with a center that provides a journey to find one's inner balance as a principle of mental structure. A compound word of “manda”, which means center and essence, and “la”, which means possession and achievement, “mandala” refers to possessing the essence in the shape of a circle. Analytic psychologist Carl Jung (Jung, C.) began to use mandala as a therapeutic technique, which has since been expanded into mandala art therapy. Jung asserted that the act of drawing within the circle facilitates relaxation and the content of the mandala allows “expression of the self-healing process” (20).

A structured mandala is an artistic activity in which a pre-designed mandala pattern is colored. Coloring a mandala is an appropriate art therapy activity to investigate its effectiveness because it involves the manipulation of art materials and individual decisions about color, shape, size, and patterning, and can produce a sense of accomplishment (21). Additionally, it has been shown to be equally effective in reducing anxiety (22–24).

Digital transformation requires structuring. It is necessary to examine the format for organizing and conducting clinical sessions to digitize the current methods of arts therapy. Choi (25) conceptualized the “session operating structure” as a clinical structure theory of arts therapy comprising “whole session constructing method (clinical period, goal, and activities)” and “single session proceeding method (progress structure and clinical environment).” The present study analyzed the structure of the existing depression-related mandala art therapy by adding new categories for the evaluation method and runtime to the session operation structure theory. Consequently, the arts therapy clinical system for digital transformation was conceptualized as the composition method of a comprehensive whole session (clinical period, goal, activity, and evaluation method) and progress method of a respective single session (progress structure, runtime, and clinical environment). This concept was used as an analytical framework to extract the major elements of the Digital Mandala.

#### 3.1.2. Five stages of digital content development process

The evidence-based digital content development process was conducted in an agile format. The Digital Mandala service was developed through preliminary research, design, development, commercialization, and advancement (Table 2).

**Table 2:** Digital content development process.

**Phase**	**Contents**
Preliminary research	Literature review for content development To collect and analyse the evidence for digital content
Design	Development of a complete roadmap for the multisensory digital content To make a proposal, storyboard, and screen design
Development	Digital content visualization Considering operation system and software patent registration
Commercialization	Support for continuous content usage and standard verification To create a manual and user management system
Advancement	Evaluate content and obtain feedback Supplement content through evaluation of utilized elements

##### 3.1.2.1. Preliminary research

In the preliminary research phase, market analysis and fundamental information were investigated, followed by a literature review to collect evidence for content development. Evidence-based digital content requires scientific, empirical, and theoretical basis from various perspectives. Therefore, three key factors of evidence-based digital content development were identified.

First, medical advice from psychiatric clinicians and mental health specialists were used throughout this process to collect clinical evidence. Furthermore, the “Evidence-based Guideline for Depression in Primary Care” issued by the Korean Academy of Medical Sciences and the Korean Disease Control and Prevention was consulted. Second, the features of the arts therapy were examined. Theoretical data on stimulating factors such as the senses of vision, hearing, and touch and arts therapy techniques (i.e., art, music, and reflective writing) were gathered. Finally, the device, gamification, and UI/UX, as well as the data assessment criteria, were determined. In contrast to digital therapeutics, the digital wellness service does not require strict clinical evidence to validate its therapeutic effect. However, it is essential to determine the empirical and theoretical evidence and secure its reliability and validity as a mental health service. In this study, a “digital transformation factor” was identified as the data factor for evaluation. Table 3 describes the key elements of the evidence-based digital content.

**Table 3:** Key factors of the evidence-based digital content.

**Key features**	**Contents**
Clinical factors	Symptoms, characteristics of content users, treatment methods, and effects Digital transformation from clinical aspects
Integrative arts therapy factors	Integrative arts therapy features Analysis of sensory elements such as sight, hearing, and touch
Digital transformation & data factors	The structure of digital content and data factor extraction Device, gamification, and UI/UX

##### 3.1.2.2. Design

In the design phase, it is necessary to define the type of data to be collected later by considering its therapeutic significance. Therefore, a data factor, a storyboard, and a screen design were proposed. It provided a visual presentation of the organization and development of the content. Moreover, it discussed the evaluation of use intention, possibility of continuous use, perceived usefulness, and satisfaction. Concerning the content, the Digital Mandala service was designed under four main themes (Table 4). First, art activities, including choosing a pattern, drawing lines, coloring a mandala, and rotating the direction, were planned. Second, provisions were made for participants to select their emotions and music. Third, a writing activity of the mandala note was designed, which consisted of creating a title and expressing their feelings experienced while creating the mandala. The final activity was to appreciate the mandala.

**Table 4:** Digital Mandala service design.

**Digital Mandala service design**					
**Mandala artwork**		**Feeling and music**		**Mandala note**		**Appreciation**
a. Selecting a mandala pattern b. Drawing lines c. Mandala pattern coloring d. Rotating the mandala's direction	→	a. Choosing a present mood b. Choosing a music	→	a. Creating a title b. Reflective writing	→	a. Closing session

##### 3.1.2.3. Development

During the development phase, it is necessary to choose a content developer while also establishing an operating system to be used after content development. Furthermore, content usage management and software patent registration should be considered. At this stage, a content developer was selected and the core technologies related to digital content production were investigated. Actual content creation proceeded with the UI/UX design, choice of data collection strategies, and establishment of an operating system. Since digital content is created through flexible interactions, agile responses from practitioners are required during the development phase. Consequently, we continued to collect evidence for production and achieved content creation.

##### 3.1.2.4. Commercialization

In the commercialization phase, it is necessary to release the developed content, create a manual that introduces functions, and create content accounts. Furthermore, the assessment data defined in the preceding stage must be gathered in real-time and saved in the Cloud to build the framework for standard verification and improvement. As part of the Seoul Metropolitan Government's public mental health services, this pilot project was commercialized implementing the Digital Mandala service for depression management. The components and core evidence of the commercial content are presented in the results.

##### 3.1.2.5. Advancement

In the advancement phase, it is important to supplement the contents by analyzing the acquired public data and user feedback. Standard verification and advancement work can be conducted by assessing the acquired log data and reviewing survey data on the use of the contents, which includes items such as the intention to use, continued use, and satisfaction as a public mental health service. Additionally, it requires supplementary work to function as high-quality content that may be utilized continuously, such as obtaining feedback on the UI/UX and progressing with upgrades. Throughout this process, digital content can establish a standardization system using quantitative data and secure scientific results, such as clinical efficacy and validation. The scope of this study is confined to the creation process and evidence-based element analysis; hence, it does not include the assessment of digital content usability or the evaluation of efficacy through pre- and post-inspection. Content advancement will become possible in the future using the digital transformation factors presented in this study.

### 3.2. The core elements of evidence-based digital integrative arts therapy

#### 3.2.1. Clinical factors for depression

##### 3.2.1.1. Depression management

Since an increasing number of contemporary people have been reporting depressive feelings and lethargy following the COVID-19 pandemic, the new phrase “COVID Blues” has been coined. This phenomenon can be regarded as a collective depression that arose with the advent of a contactless era of social distancing. The Korean Ministry of Health and Welfare, in an attempt to provide necessary psychological support services for Korean citizens, has been conducting the “COVID-19 National Mental Health Survey” on a quarterly basis since 2020 to comprehend the state of their citizens' mental health. According to the statistics in the second quarter of 2022, even though the index of the depression risk group improved (22.8% in March 2021 to 16.9% in June 2022) after social distancing ended, the figure still represented a high and dangerous level that was over five times higher than that of 2019 (3.2%) before the COVID-19 outbreak (26). Concurrently, the rate of suicidal ideation (12.7% in 2022) also increased to nearly three times higher than that in 2019 (4.6%). Particularly, the depression score and ratio of depression risk groups among people in their 30s were consistently higher than those in other age groups, indicating the importance of supportive mental healthcare services for young people in the post-COVID-19 era.

Major depressive disorder, commonly known as depression, is a mental health problem defined as a lethargic state accompanied by a chronically depressed mood or sad state and loss of interest or pleasure in life. It is classified as a very serious mental disease because it is associated with anxiety and sleep difficulties, deteriorates interpersonal competency and occupational ability, and can lead to suicide in extreme situations. The causes and risk factors for depression differ from individual to individual; they are most typically explained by the stress vulnerability model (27). In other words, emotionally vulnerable people may suffer from depression more easily if they are exposed to stressful life events in their daily lives. The Diagnostic and Statistical Manual of Mental Disorders (DSM-5) and the Patient Health Questionnaire (PHQ-9) were used to assess the severity of depression, and pharmacological or non-pharmacological treatments were provided (28). Psychological and psychosocial interventions, such as acceptance and commitment therapy (ACT) and cognitive behavioral therapy (CBT), are preferred for mild or moderate depression and have effects similar to pharmacological treatments (27, 29–32). Digital interventions could significantly contribute in expanding mental healthcare and reducing the considerable undersupply of mental disorders or psychological treatments worldwide (33). Previous studies have indicated that digital mental health interventions help relieve depressive symptoms (6, 34–37).

##### 3.2.1.2. The evidence of mandala art therapy

Although large-scale studies specifically examining mandala art therapy are lacking, some studies included in Table 5 above show that structured mandala art therapy can have a positive impact on emotions such as depression, anxiety, stress reduction, and relaxation. However, it is important to recognize that the existing evidence for mandala coloring is based case reports, qualitative studies, and small exploratory studies. More high-quality studies, such as randomized controlled trials and large-scale studies, are required to establish stronger evidence for the specific therapeutic effects of structured mandala coloring. This study's digitalization of mandala art therapy will supplement the limitations of evaluating the efficacy of existing art therapy techniques. In addition, it is expected that substantial results can be obtained by analyzing detailed factors of digital integrative arts therapy, going beyond the current evaluation method that tests for pre- and post-treatment differences.

**Table 5:** The effects of mandala art therapy.

**References**	**Target disease/condition**	**Number of participants**	**Results and effects**	**Effect sizes**
Gurcan and Turan (38)	Anxiety & depression in hospitalized adolescents with cancer	60	To improve anxiety and depression	Anxiety: large (η^2^ = 0.24) Depression: large (η^2^ = 0.24)
Carsely and Heath (39)	Test anxiety	167	To decrease the level of test anxiety	Large (partial η^2^ = 0.14)
Bi and Liu (40)	Social anxiety	72	To decrease social anxiety	Large (partial η^2^ = 0.34) *Post-hoc*: mandala > free drawing, control
Pisarik and Larson (41)	Authenticity, wellbeing	68	To strength self-awareness, unbiased processing, personal growth, and self-acceptance	Awareness: large (partial η^2^ = 0.47) Unbiased processing: medium (partial η^2^ = 0.08) Personal growth: large (partial η^2^ = 0.52) Self-acceptance: small (partial η^2^ = 0.05)
Curry and Kasser (23)	Anxiety	84	To reduce the level of anxiety	Effect size is not suggested, but difference between groups is significant.
Khademi et al. (42)	Anxiety in hospitalized COVID-19 patients	70	To reduce the level of anxiety	Effect size is not suggested, but difference between groups is significant.
Lee and Ryu (43)	Emotional stability, attentiveness among	30	To increase emotionality, especially, satisfaction and self-confidence.	Effect size is not suggested, but difference between groups is significant.
Babouchkina and Robbins (44)	Negative mood	67	To reduce the level of negative affect	Effect size is not suggested, but difference between groups is significant.
Kim et al. (45)	Subjective wellbeing, resilience, hope	28	Only hope increased	Effect size is not suggested, but difference between groups is significant.

#### 3.2.2. Integrative arts therapy features

The Digital Mandala is an integrative arts therapy content that stimulates the senses of sight, hearing, and touch through a digital experience. This content, which combines art, music, and literary interventions, aims to increase therapeutic efficacy by balancing the states of closeness and separation. Owing to the characteristics of the mobile device, tactile stimulation felt by the hand and visual stimulation of images are continuously activated during the screen-touch interaction process. Furthermore, the Digital Mandala content is accompanied by auditory stimulation via emotional music. Table 6 lists the specific features of the Digital Mandala.

**Table 6:** Digital Mandala as an integrative arts therapy.

**The stage of Digital Mandala service**	**Projective model**	**Sensory stimulus**	**Activity type**	**Ultimate achievement**
Mandala artwork	Art—drawing and painting of a structured mandala	Sight, touch	Expression—closeness, immersion, and under-distance	Catharsis—finding an aesthetic distance between closeness and separation through various stimuli and activities using arts
Feeling & music	Music—to express the current mood through melody	Hearing		
Mandala note	Literature—reflective journal writing	Touch		
Appreciation	Integration for achieving balance	Multi-sensory	Appreciation—separation and over-distance	

##### 3.2.2.1. Mandala artwork

The human desire to select colors occurs in the brain. The brain stimulation through colors is expressed as emotion, which is a physical instinct. Each color has its own vibration and frequency that stimulates a response in the process of being transmitted to the brain and creates emotional expression. When a specific color is transmitted to the hypothalamus through visual perception, it passes through the A10 nerve and produces the judgment of good or bad. Therefore, choosing a color can be perceived as a measurement of not only one's desire for pleasure but also as a tool to detect internal changes (46). A study on color preferences based on depression and anxiety levels showed that the group with high levels of depression preferred achromatic colors the most, while the group with high levels of both depression and anxiety had the highest preference for cold colors (47). Alternatively, the group with low depression and high anxiety preferred warm colors, whereas the group with both low depression and anxiety preferred neutral colors. Thus, colors have a deep relationship with psychological elements, and expressing one's emotions with lines and colors to experience purification represents the therapeutic properties of art.

**Figure 2:**
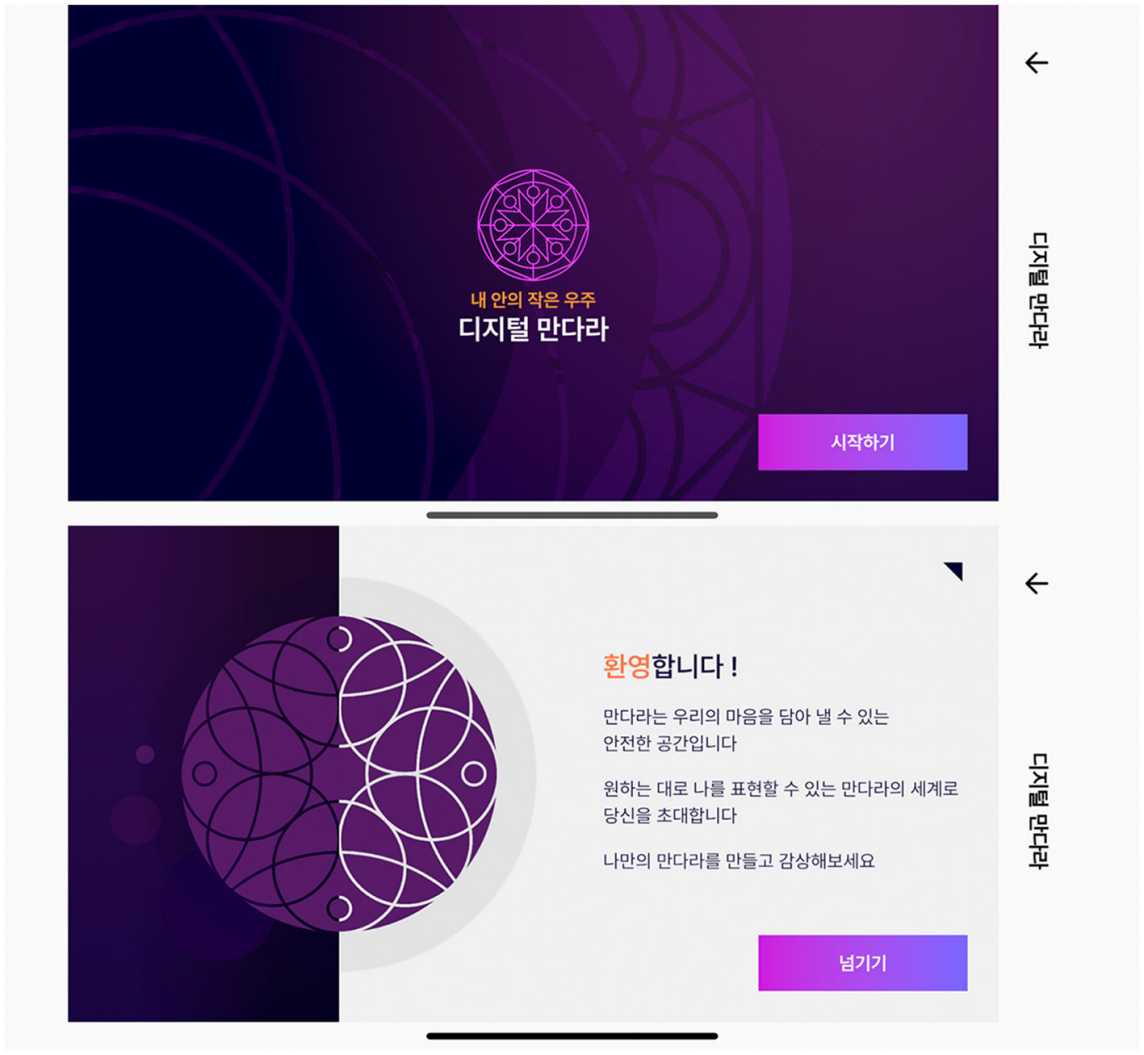
Digital Mandala service's splash screen and a tutorial.

In art therapy using the structured mandala, such tendencies of clients can be analyzed according to the direction of their coloring. In the mandala artwork, introverts unconsciously tend to color from the center to the circumference of the circle, whereas extroverts or those who currently feel nervous, powerless, exhausted, have low concentration, and high distractibility tend to color the mandala from the circumference into the center (48). Additionally, since the mandala artwork of improvisational expression is an unconscious activity in which participants' emotions are reflected in shapes or colors, artistic psychological elements, such as color symbolism and shape psychology, can function as factors that can be evaluated therapeutically. Figure 2 illustrates the Digital Mandala's splash screen and a tutorial.

**Figure 3:**
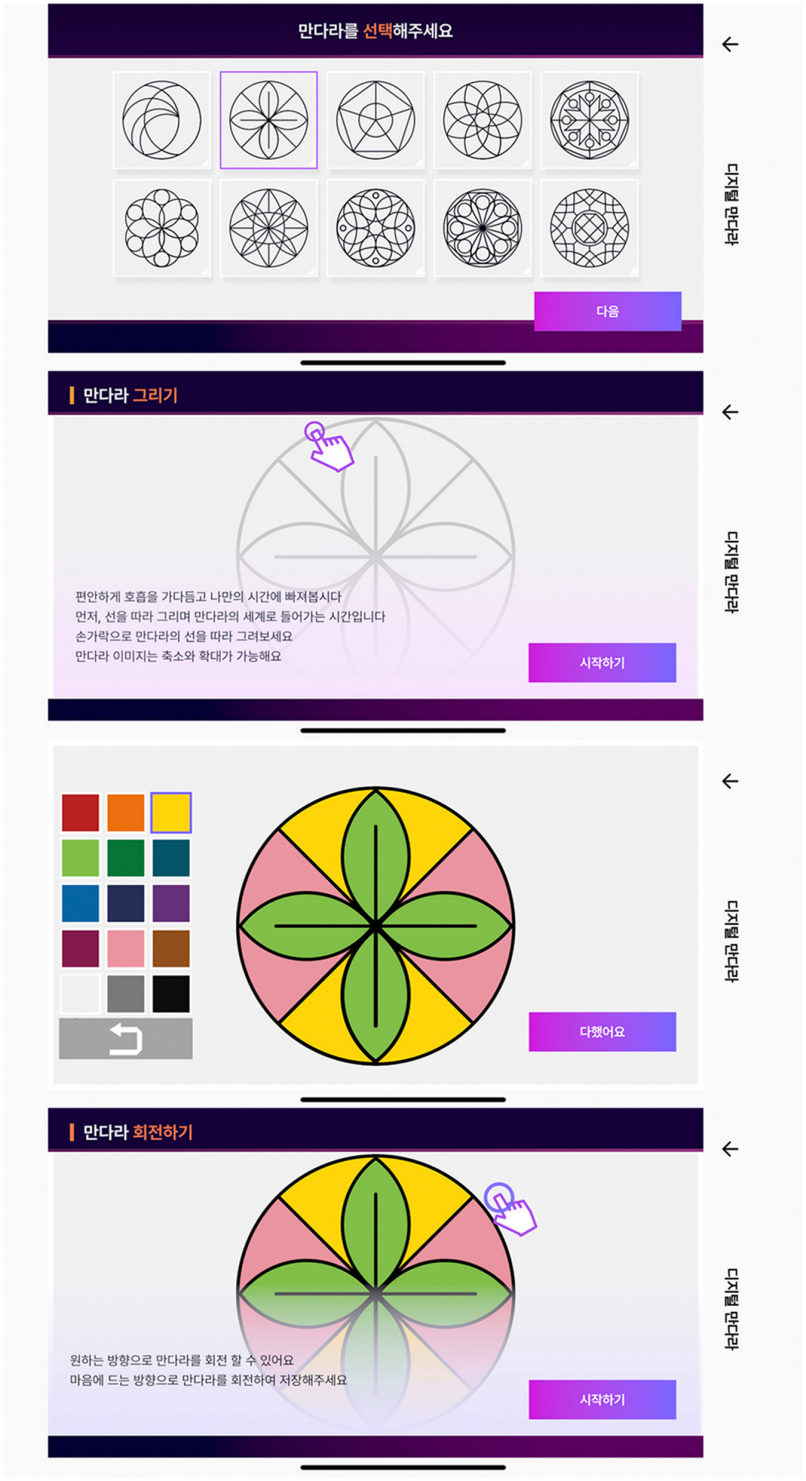
The sequential process of mandala artwork phase.

In the Digital Mandala service, the mandala artwork process includes a structured mandala pattern selection, line drawing, and coloring, followed by a rotation in the appropriate direction (Figure 3). The structured mandala contains 10 patterns: simple, normal, and intricate for complex patterns. Fifteen colors were chosen based on the sRGB values provided by the Korea Industry Standard (KIS). The colors consist of 12 chromatic and three achromatic colors.

- **Chromatic colors:** red, yellow-red, yellow, red-purple, green-yellow, green-purple, pink, brown, blue-green, blue, and purple-blue
- **Achromatic colors**: white, gray, and black.

##### 3.2.2.2. Feeling and music

Music has been used as a sensory tool to express people's mood and emotions. Dynamic experiences, such as rhythm and melody, provided in the process of integrative arts therapy stimulate individual emotions and expand conscious awareness (49). Thus far, diverse studies have investigated the recognition and classification of emotions associated with musical components. Their representative results have shown that fast tempo music produces happiness or excitement, while slow tempo music may be associated with sadness. Additionally, low tones represent dark and sad circumstances (50–55).

Robert Thayer (Thayer, R.), a music psychologist, defined a two-dimensional mood/atmosphere model by setting “Stress” and “Energy” categories. Here, “Stress” is the degree from “Happy” to “Anxious/Sad,” while “Energy” is the degree from “Calm” to “Energetic.” The mood model classifies the emotions of “Exuberant, Anxious/Frantic, Contentment, Depression” within the two axes of “Stress” and “Energy” (56). When acoustic characteristics such as intensity, timbre, pitch, and rhythm of music act as parameters, they can affect the listener's mood and emotions, which can then work as factors that create the atmosphere of music [Table 7; (50)]. In this manner, music works as a medium that affects emotions and contains a sensory function for emotional expression.

**Table 7:** Moods classified in accordance to audio features.

**Mood**	**Intensity**	**Timbre**	**Pitch**	**Rhythm**
Happy	Medium	Medium	Very high	Very high
Exuberance	High	Medium	High	High
Energetic	Very high	Medium	Medium	High
Frantic	High	Very high	Low	Very high
Sad	Medium	Very low	Very low	Low
Depression	Low	Low	Low	Low
Calm	Very low	Very low	Medium	Very low
Contentment	Low	Low	High	Low

**Figure 4:**
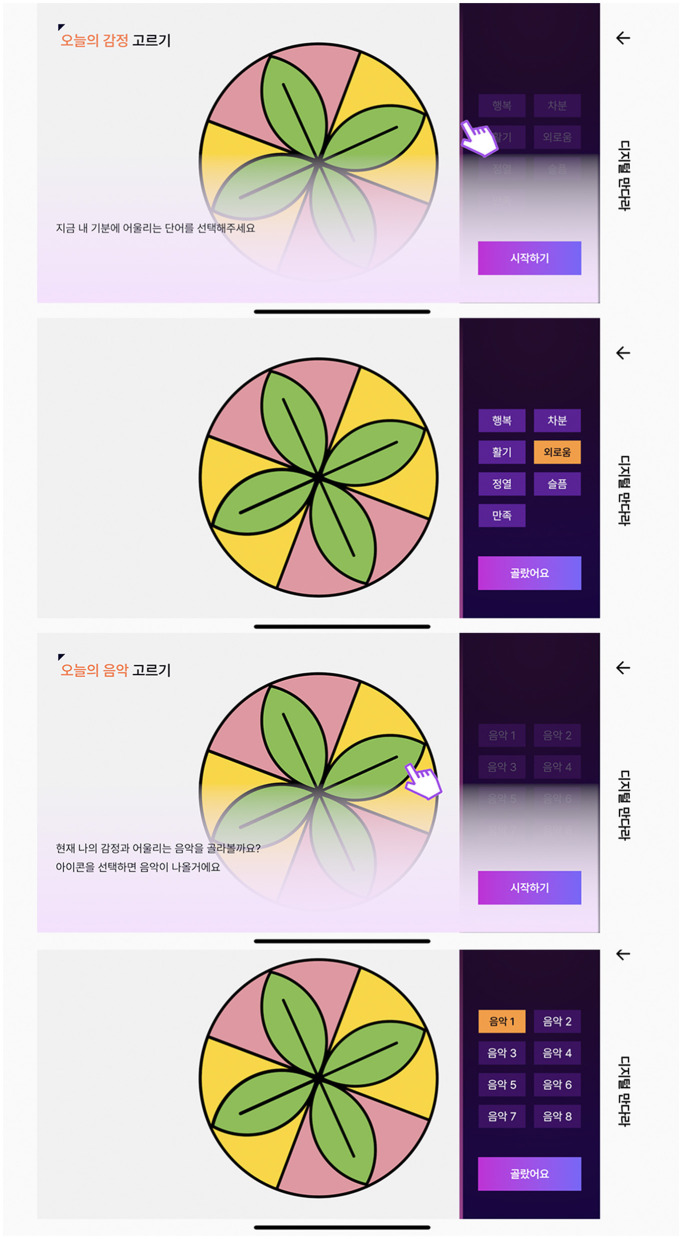
Feeling and music selection phase implementing screen.

The second stage is to choose a feeling and emotional music that represents the user's current condition (Figure 4). The purpose of this stage is to help participants recognize their state of mind at a given moment by exploring their current emotions. Their emotions can be musically expressed by selecting and appreciating music techniques within the projective model of integrative arts therapy. The seven types of emotions comprised “happy, exuberance, energetic, sad, depression, calm, and contentment”. Furthermore, “depression” was added to the seven emotions listed above and eight different sound sources with distinct styles were provided as music options. However, it was provided by hiding the emotion labels.

- **Feeling:** happy, exuberance, energetic, contentment calm, loneliness, and sad
- **Emotional music:** music 1 (happy), music 2 (exuberance), music 3 (energetic), music 4 (contentment), music 5 (calm), music 6 (loneliness), music 7 (sad), and music 8 (depression).

##### 3.2.2.3. Mandala note

**Figure 5:**
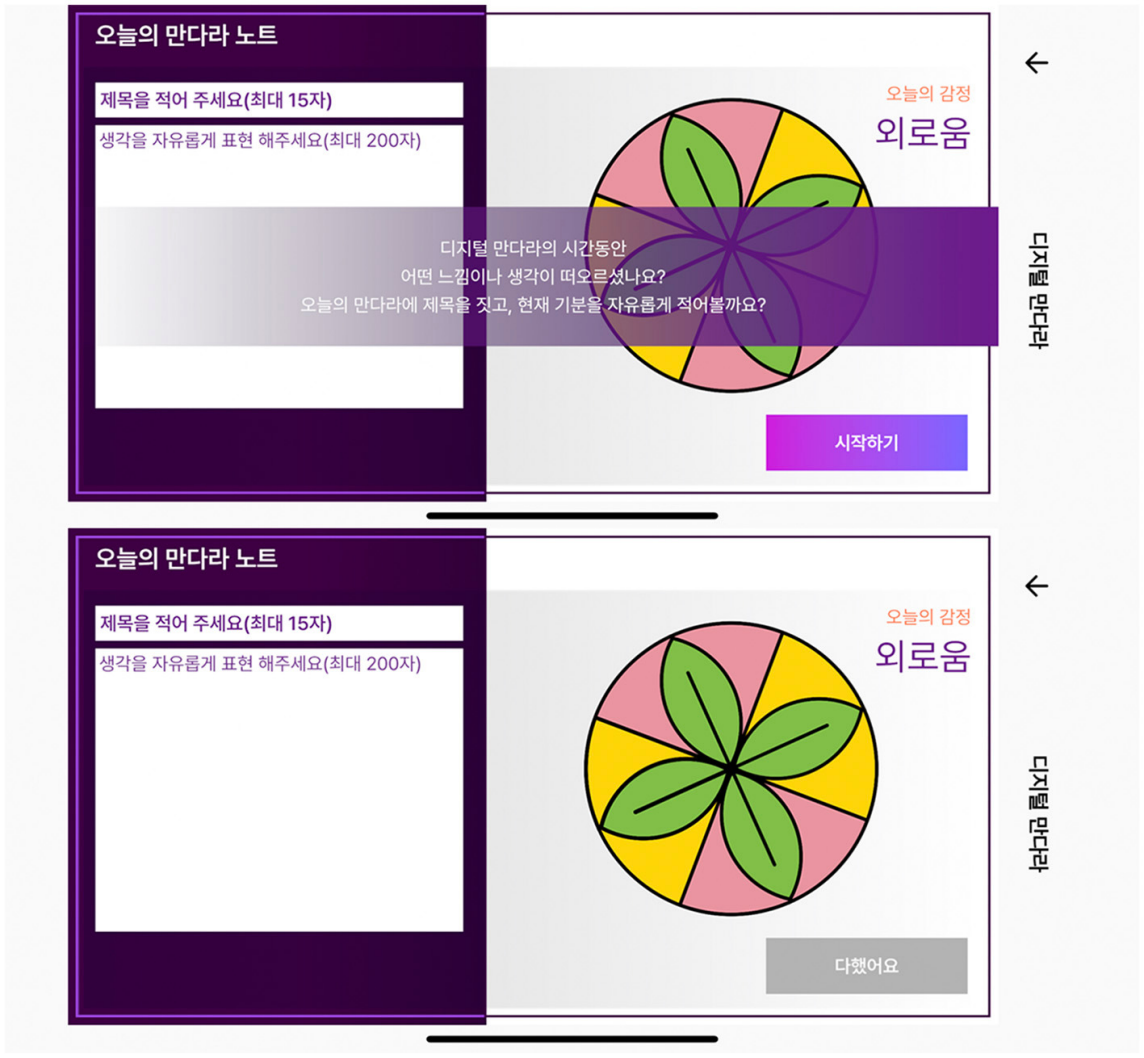
Mandala note for creating a title and reflection writing.

The use of literary media allows the linguistic imagery of one's inner world to be expressed through symbols and metaphors. This has a therapeutic effect that encourages the reorganization of memories and psychological dissipation as a way to express thoughts and feelings (49). Reflective writing skills allowed participants to verbalize their current feelings and experiences. Introspective writing in the mandala note stage aims to allow participants to consciously write a title for the mandala on which they have worked and verbalize their current feelings and thoughts to help them express their unconsciousness (Figure 5). This therapeutic practice is distinct from the simple mandala coloring activity.

##### 3.2.2.4. Appreciation

**Figure 6:**
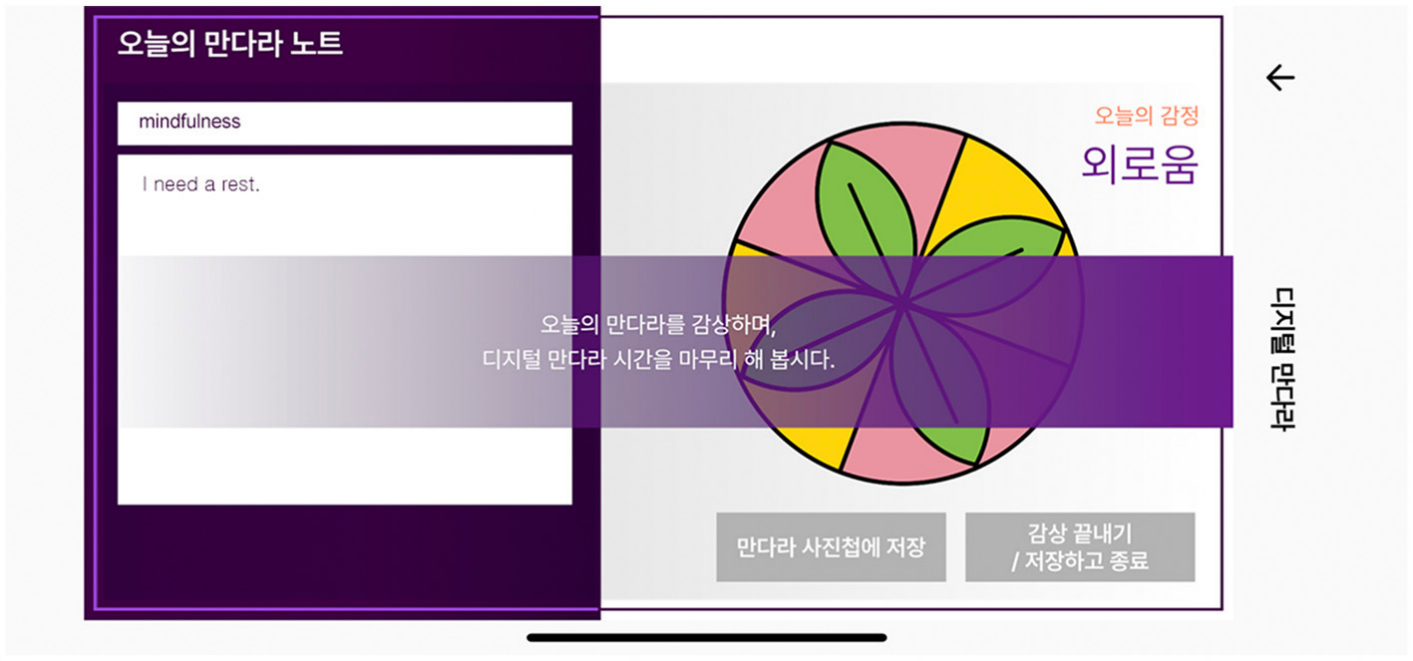
Appreciation implementing screen.

Maintaining a balance between the two states of separation and closeness is referred to as “distancing” (57). According to the aesthetic distance theory, catharsis occurs at the balance point between over- and under-distance. Expressive activities are creative, which serve to emphasize the intrapsychic elements during the experience of immersion, and appreciation becomes a cognitive process that regards one's work objectively. When participants complete all the expressive activities, they are then led to the appreciation stage, which creates an emotional distance that was once closely attached to them. The purpose of the appreciation stage is to internalize the arts therapy experience by showing all outcomes in an integrated manner (Figure 6). This forms an aesthetic distance, which is the midpoint between closeness and separation. Additionally, it helps participants gain insight and self-awareness by maintaining a reasonable distance from a therapeutically immersive environment.

#### 3.2.3. Data factors for digital transformation

##### 3.2.3.1. The structure of the digital mandala

The Digital Mandala service was digitalized from the existing practice of immersive mandala art therapy into a mobile app. It was developed as an evidence-based digital content, including three types of evidence (i.e., clinical elements, integrative arts therapy features, and data factors for digital transformation). The session operation structure to digitize mandalas was elucidated using the theory of the arts therapy clinical system (Table 8). The structure of the Digital Mandala was divided into comprehensive whole session and respective single sessions as a concept. The former includes the clinical period, goal, clinical activities, and evaluation method, while the latter comprises the progress structure, runtime, and clinical environment.

**Table 8:** The structure of Digital Mandala.

**Session structure system**			**Traditional method**		**Digital transformation**
Comprehensive whole session	Clinical period	→	12–16 session	→	Personalized
Goal			Reduce symptoms of depression and anxiety Mindfulness practices and self-expression through art therapy activity Relaxation and mood improvement		Self-management of psychological conditions using digital content in daily life Same as the traditional method of mindfulness, relaxation, consciousness
Clinical activity			Structured mandala coloring art therapy technique		Digital mandala coloring art therapy software
Evaluation method			Quantitative evaluation via pre-post scale screening test (DSM-5, PHQ-9, CES-D-10, etc.) Qualitative evaluation via client's expression and activity analysis (color, symbolic meaning, etc.)		Digital self-screening test (PHQ-9, CES-D-10, GAD-7, ISI-K4) Data factor quantitative evaluation Large-scale trials available
Respective single session	Progress structure		Introduction, activity, reflection, and closure Mandala pattern choosing and coloring		4 steps (art working, music & feeling, mandala note, and appreciation)
	Runtime		60–90 min		Personalized
	Clinical environment		A safe and supportive environment		Anywhere at any time Digital wellness service (Smartphone app)

The traditional method and digitized content share the same objectives and activities because they both attempt to induce mindfulness through the mandala coloring technique, which aims to reduce depression and anxiety. However, the Digital Mandala is distinct in that its users can access it anywhere at any time and self-manage their depression due to the nature of mobile devices. Additionally, the digital content has its own unique evaluation methods. Traditional arts therapy mainly uses qualitative evaluation (i.e., analysis of clients' expressions, changes, symbols, and metaphors), which has been criticized on its lack of clinical effectiveness and limitations in effectiveness verification. Despite continuous attempts to examine the degree of change through pre-post assessments, they have not gained sufficient support for clinical effectiveness due to small sample sizes and biased results. The limitations of traditional evaluation methods can be overcome through digital transformation. Because digital content is easy to access, large-scale pre-post assessments can be performed to validate its effectiveness. Furthermore, the data factor of mandala art therapy can serve as a criterion for quantitative evaluation.

##### 3.2.3.2. The extraction of data factors

The extraction of data factors is fundamental for digital transformation. Data factors serve important functions throughout the content development procedure. Digital mental healthcare content is based on clinical evidence and permits quantitative data analysis with therapeutic value. Therefore, the present study established a strategy to extract data factors of the evidence-based digital content and apply them as evaluation elements to remedy the limitations inherent in traditional evaluation methods. Table 9 lists the data factors derived as key elements for digital transformation.

**Table 9:** Data factors of Digital Mandala.

**Classification**	**Content component**		**Data factors for evaluation indicators**
Art	Mandala artwork	Selecting a pattern of mandala	The type of mandala pattern (simple to complex)
		Drawing lines of mandala	Touch position data (drawing direction)
		Mandala pattern coloring	The type and number of colors (variety of colors) The frequency of colors Touch data for each cell of the pattern The number of filled squares at the end of coloring The number of revisions Run-time data of coloring (degree of immersion)
		Rotating the mandala's direction	Rotation direction (rotation angle)
Music	Feeling and music	Choosing a present mood	The type of emotion (0–6)
		Choosing an emotional music	The type of melody (0–7)
Literature	Mandala note	Creating a title	The meaning of the mandala's title
		Reflective journal writing	The content and meaning of the journal To review positive, negative, informative words, or contexts
Integration	Appreciation	Closing session	Run-time data of all contents

Regarding data extraction, the type of mandala, touch position, diversity and frequency of colors, touch data for filled spaces at the end of coloring, rotation direction, and amount of time can be collected in the mandala artwork phase. Additionally, it is possible to evaluate emotions based on depressive symptoms and the mood component of music, as well as to analyse language data obtained from the mandala-note stage. Future studies should evaluate data systematically to provide individualized services. The log data collected at each stage and survey data on usability should be analyzed to assess the service. This provides a standard for customizing and upgrading the content.

## 4. Limitation and future scope

This manuscript details the development of digital integrative arts therapy content for public mental health services and focuses on expanding opportunities for leveraging digital in the future of mental health care. The scope of this study is limited to providing a practical protocol that includes an evidence-based element for developing the “Digital Mandala” service. It is meaningful to describe in detail the process that shows how the conversion of structured mandala from traditional use to app was achieved, but it does not include the application results of the Digital Mandala services.

Based on the data factors found in this study, it will be possible to create an evaluation data set of digital integrative arts therapy content for managing depression. The living lab and open innovation system can also be used more by analyzing large-scale public data. The public mental health data can be analyzed through artificial intelligence technology, which is expected to be used as a basis for deriving significant results in a new form, going further than the existing evaluation method. Future studies need to be conducted to verify its efficacy, effectiveness, and efficiency through large-scale data collection and analysis of correlations between depressive symptoms and arts therapy factors. Especially the arts factors, according to the degree of depression, should be analyzed and evaluated quantitatively.

## 5. Discussion and conclusions

This study was conducted to lay the groundwork for digital transformation in the field of art therapy for the public mental health services and to explore its possibilities. A practicable protocol for the development of evidence-based digital integrative arts therapy was presented, which converted the traditional structured mandala coloring technique into a mobile app called “Digital Mandala”. The study findings have been summarized below.

First, digital transformation requires structuring. Digital content for integrative arts therapy can be structured based on the “session operating structure” theory (25). The clinical system of arts therapy comprises the composition method of a comprehensive whole session (clinical period, goal, activity, and evaluation method) and the progress method of a respective single session (progress structure, runtime, and clinical environment). Second, evidence-based digital content can be developed through five stages of the virtuous cycle development process that include preliminary research, design, development, commercialization, and advancement. Third, the main features of the evidence-based digital content include “clinical evidence”, “arts therapy features”, and “data factors”, which might serve as criteria for content production and evaluation. There is a need to collect evidence that explains the effects of the existing method and the detailed scene-specific components of digital content. It is important to provide a specific justification for the composition of the content and to clarify the significance embedded within each element. Fourth, the fundamentals of digital transformation include the conversion of the traditional operation method into a digital format to develop a standardized system that collects, analyzes, and processes quantitative data. Digital conversion through data factor extraction enables the quantitative verification of the effectiveness of arts therapy and can serve as an evaluation criterion for the evidence-based digital content. Consequently, the Digital Mandala implemented based on this research has been provided as an integrating arts therapy content that encourages creative self-expression and the healing process among participants in order to prevent and manage depressive symptoms. This content is valuable as a digital service that stimulates multi-sensory and provides expression and appreciation by utilizing various media such as art, music, and literature.

The significance of this study is that it provides a development protocol for data-based customized services through the Digital Mandala, which digitally converts the existing experiential approach of art therapy into digital content. Additionally, it presents the possibility of verifying the effectiveness of art therapy in the future by deriving data factors and digitally implementing the mandala technique. The data collected through the Digital Mandala can be used to expand the range of traditional arts therapies that have lacked quantitative or scientific effectiveness verification in randomized controlled trials (RCTs). If it is evaluated quantitatively using data elements, it will not only function as digital wellness content but also as digital therapeutics. Through this study, it is expected that the specific utilization and spread of digital and arts convergence content as public mental health services will be achieved.

## Data availability statement

The original contributions presented in the study are included in the article/supplementary material, further inquiries can be directed to the corresponding author.

## Ethics statement

Ethical approval was not required for the studies involving humans because this study aims to present a practical protocol for the development of digital mental health services. The studies were conducted in accordance with the local legislation and institutional requirements. Written informed consent for participation was not required from the participants or the participants' legal guardians/next of kin in accordance with the national legislation and institutional requirements because this study does not present the applied results of digital services.

## Author contributions

HK participated in the digital healthcare expert. YC contributed to the integrative arts therapy expert, and they conducted convergence research. HK and YC conceptualized, designed, and implemented the study. YC contributed to the original draft's writing, literature review, and analysis of the results. HK contributed to the project administration, supervision, methodology, and validation. All authors have discussed and agreed to the published version of the manuscript.
